# Environmental surface testing for severe acute respiratory syndrome coronavirus 2 (SARS-CoV-2) during prolonged isolation of an asymptomatic carrier

**DOI:** 10.1017/ice.2020.300

**Published:** 2020-06-16

**Authors:** Kyeong Seob Shin, Hee Sue Park, Jisu Lee, Joon Kee Lee

**Affiliations:** 1 Department of Laboratory Medicine, Chungbuk National University Hospital, Cheongju, Korea; 2 Department of Laboratory Medicine, Chungbuk National University College of Medicine, Cheongju, Korea; 3 Department of Pediatrics, Chungbuk National University Hospital, Cheongju, Korea; 4 Department of Infection Control and Prevention, Chungbuk National University Hospital, Cheongju, Korea

## Abstract

Environmental surface testing was performed to search for evidence of severe acute respiratory coronavirus virus 2 (SARS-CoV-2) environmental contamination by an asymptomatic SARS-CoV-2 carrier with persistently high viral loads under isolation. No evidence of environmental contamination was found. Further studies are needed to measure environmental contamination by SARS-CoV-2 carriers and to determine reasonable isolation periods.

The transmission dynamics of severe acute respiratory syndrome coronavirus 2 (SARS-CoV-2) remain unclear. Some individuals are asymptomatic carriers of SARS-CoV-2, and some patients who have recovered from coronavirus disease 2019 (COVID-19) may continue to carry SARS-CoV-2.^1^ The relationship between the presence of viral RNA in the nasopharynx and ongoing contagiousness remains unclear.^2^ A better understanding of the role of asymptomatic carriers is needed to inform isolation policies and public health measures to prevent the spread of SARS-CoV-2. Extensive environmental contamination by a symptomatic COVID-19 patient has been reported,^3^ but data regarding environmental contamination by asymptomatic SARS-CoV-2 carriers are limited. The purpose of this study was to search for evidence of environmental contamination by an asymptomatic SARS-CoV-2 carrier with persistently high viral loads.

## Methods

### Case description

On April 5, 2020, a 44-year-old mother and her 11-year-old daughter were referred from a community treatment center (CTC) to Chungbuk National University Hospital, an 810-bed designated COVID-19 referral hospital in Cheongju, Korea, because the mother was experiencing a sore throat which had started the previous day. The CTC is a novel institution that manages patients with mild COVID-19 or asymptomatic SARS-CoV-2 infection or carriage using out-of-hospital isolation.^4^ The mother and her daughter had been diagnosed with COVID-19 on March 9 and March 12, respectively, during a mass screening conducted in response to a large outbreak in Daegu Metropolitan City, Korea, but neither of them had experienced any symptoms of COVID-19, such as fever, respiratory/gastrointestinal symptoms, anosmia, or dysgeusia, until the mother developed a sore throat on April 3. The daughter remained asymptomatic, but because she was not old enough to be isolated on her own at the CTC, she was referred with her mother and they shared a room. Chest radiography of both patients on admission did not reveal any sign of infiltration of the lungs or any focal lesions. The mother was released from isolation on April 23. Her daughter was transferred to the Daegu Medical Center on April 24 and was discharged on May 13 after 62 days of isolation having experienced no symptoms during the entire isolation period.

### Detection of SARS-CoV-2

The Korea Center for Diseases Control and Prevention (KCDC) requires 2 negative real-time reverse-transcription polymerase chain reaction (RT-PCR) tests for SARS-CoV-2 using samples collected at least 24 hours apart as a minimum requirement for releasing individuals who are diagnosed with SARS-CoV-2 infection. Nasopharyngeal swabs were collected approximately every 5–7 days if the test was positive, based on shared decision making between the physician and the mother and her daughter. If the test was negative, it was repeated the following day.

RT-PCR was performed in house using a SARS-CoV-2 test kit (Allplex 2019-nCoV Assay, Seegene, Seoul, Korea). The assay simultaneously detects 3 target genes of SARS-CoV-2. A cycle threshold (Ct) value <40 is reported as positive for each gene. The Ct refers to the number of cycles required for the fluorescent signal to cross the threshold in RT-PCR, so a lower cycle threshold value indicates a higher viral load. Positive and negative results were reported when all 3 genes were positive or negative, respectively, and the result was reported as inconclusive if the test is positive for 1 or 2 genes.

### Surface sampling and room cleaning

**Fig. 1.**
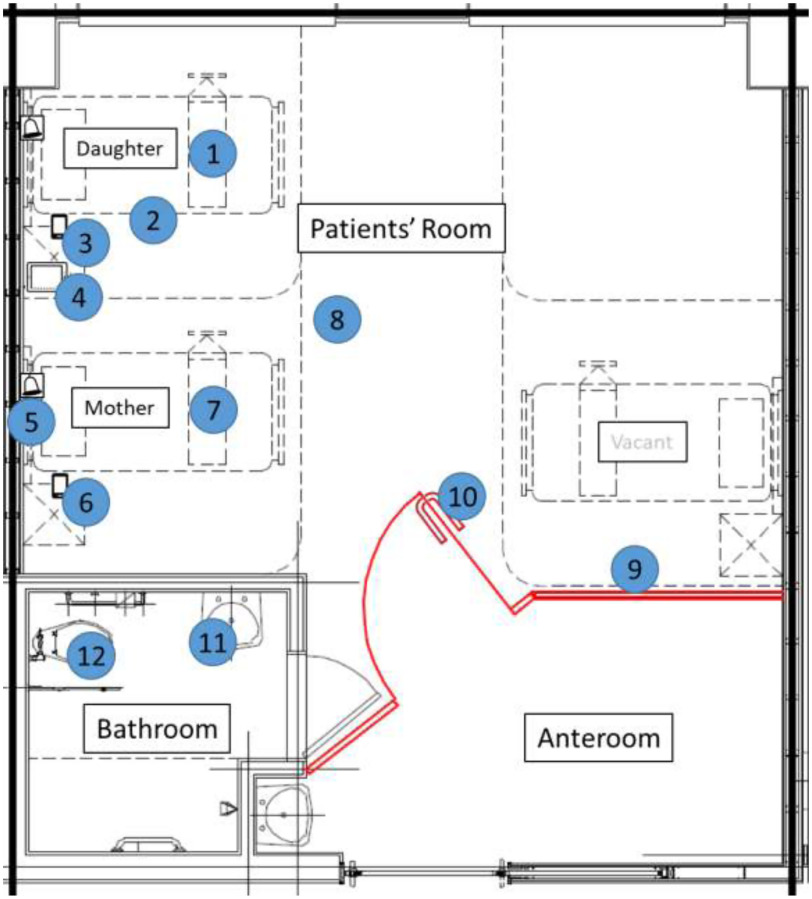
Room layout showing environmental sampling sites. Numbers are labeled corresponding to environmental sampling sites: 1, bedside table (daughter); 2, entire length of bed rail (daughter); 3, mobile phone (daughter); 4, tablet (daughter); 5, call bell attached to bed (mother); 6, mobile phone (mother); 7, bedside table (mother); 8, floor; 9, in room wall panel; 10, door handle; 11, sink (bathroom); 12, toilet bowel.

On April 22, 41 days after the initial diagnosis of SARS-CoV-2 infection (carriage) in the daughter, we tested environmental samples collected from 12 surfaces of the surrounding environment (Fig. 1). All the surfaces, except for mobile devices, were swabbed twice rigorously over almost 100% of the item or area (except for the floor), using separate sterile swabs moistened with distilled water. The mobile devices were swabbed once each on the front and the rear surfaces. Two swabs were placed together in each viral transport medium.

The room and bathroom were cleaned weekly. The most recent cleaning had occurred 4 days prior to environmental sample collection.

### Ethical considerations

The study was approved by the institutional review board of the Chungbuk National University Hospital (IRB no. 2020-04-022). Informed consent for the publication of this report was obtained from both patients.

## Results

On April 22 and 23, the test was negative in the mother, but the test was still positive in the daughter on April 22, with no decrease in the viral load (Table 1).

**Table 1.** SARS-CoV-2 reverse transcriptase polymerase chain reaction results and corresponding cycle threshold* value of the patients

	Gene	Ct value of RT-PCR from nasopharyngeal swab					
		April 5	April 10	April 15	April 16	April 22	April 23
Daughter	*E*	27.97	28.16	N/A	28.48	27.00	N/A
	*RdRp*	29.92	29.78	N/A	30.22	28.83	N/A
	*N*	30.11	30.15	N/A	30.33	29.93	N/A
Mother	*E*	N/A	N	N	N	N	N
	*RdRp*	N/A	N	N	N	N	N
	*N*	N/A	39.78	38.67	N	N	N

RT-PCR, reverse transcriptase polymerase chain reaction; N, negative; N/A, not available (no test); *RdRp*: RNA-dependent RNA polymerase.

* Cycle threshold (Ct) refers to the number of cycles required for the fluorescent signal to cross the threshold in RT-PCR; a lower cycle threshold value indicates a higher viral load.

The results of the tests on all the environmental surface samples were negative: mobile phones (both), tablet (daughter), bedside tables (both), entire length of bed rail (daughter), call bell attached to the bed (mother), wall panel/door handle (in room), floor, and sink/toilet bowl (bathroom). Most of the surfaces including the floor (epoxy) were plastic except for the bed rail, door handle (stainless steel), and sink/toilet bowl (ceramic).

## Discussion

SARS-CoV-2 is known to be transmitted primarily by respiratory droplets, but transmission by fomites is possible. A recent study which investigated the aerosol and surface stability of SARS-CoV-2 revealed that aerosol and fomite transmission of SARS-CoV-2 is plausible because the virus can remain viable and infectious in aerosols for hours and on surfaces up to days.^5^ In addition, recent studies have shown significant environmental contamination by patients with SARS-CoV-2 through respiratory droplets and fecal shedding, adding concern to the possibility of transmission by fomites.^3,6^

The degree of transmission by asymptomatic patients has drawn considerable attention because completely inhibiting transmission is critical in controlling the pandemic.^1^ Notably, the viral load levels detected in asymptomatic patients are similar to those found in symptomatic patients, which suggests the transmission potential of asymptomatic patients.^2^ Therefore, the degree and extent of environmental contamination by asymptomatic SARS-CoV-2 carrier is critical in the management of such individuals.

We describe an adolescent with >62 days of SARS-CoV-2 carriage with no symptoms during the entire period. On the day of surface testing, she continued to have a persistently high SARS-CoV-2 viral load. Nevertheless, evidence of environmental contamination was lacking even on her mobile device and tablet, 2 of her most intimate possessions. Our result is discrepant to a report of environmental contamination by an asymptomatic infant,^7^ and we believe that differences in the dynamics of transmission may exist and further investigation is needed to explore this specific issue.

Even though we made our best efforts to detect the virus, it is possible that our method may not have detected the virus (including the absence of viral culture). Nevertheless, the sample was processed using the same procedure as that used for the patient who tested positive on the day of the sample tests; thus, we believe that the possibility of false-negative detection of the virus despite its presence is low. The generalizability of the result is limited because the study is based on an observation of a single case pair, and limited information was available about the hygiene practices of the mother and daughter.

Overall, no evidence of environmental contamination was found from a pediatric patient under isolation with high viral loads. Further studies are needed to measure the extent of environmental contamination and to decide on reasonable isolation periods.
